# miRNAs-mediated overexpression of Periostin is correlated with poor prognosis and immune infiltration in lung squamous cell carcinoma

**DOI:** 10.18632/aging.204056

**Published:** 2022-05-04

**Authors:** Xu Bai, Hui Chen, Brian G. Oliver

**Affiliations:** 1 School of Life Sciences, Faculty of Science, University of Technology Sydney, Sydney, NSW 2007, Australia; 2 Respiratory Cellular and Molecular Biology, Woolcock Institute of Medical Research, Sydney, NSW 2037, Australia

**Keywords:** POSTN, micRNA, prognosis, immune infiltration, LUSC

## Abstract

Lung cancer is one of the most common malignancies with a high mortality rate worldwide. POSTN has been shown to be strongly correlated with the poor prognosis of lung cancer patients. However, the function and mechanism of action of POSTN in lung cancer remain unclear. Here, we carried out a pan-cancer analysis to assess the clinical prognostic value of POSTN based on the TCGA, TIMER, Oncomine, Kaplan-Meier, and UALCAN databases. We found that upregulated POSTN can be a promising biomarker to predict the prognosis of patients with lung cancer. High levels of POSTN correlated with immune cell infiltration in lung cancer, especially lung squamous cell carcinoma (LUSC), which was further confirmed based on the results from the TISIDB database. Moreover, the expression analysis, correlation analysis, and survival analysis revealed that POSTN-targeted miRNAs, downregulation of has-miR-144-3p and has-miR-30e-3p, were significantly linked to poor prognosis in patients with LUSC. Taken together, we identified that POSTN can act as a novel biomarker for determining the prognosis related to immune infiltration in patients with LUSC and deserves further research.

## INTRODUCTION

Air pollution is now the fourth-largest cause of premature death [1]. Urbanisation and increased population density have increased the exposure to traffic-related air pollution (TRAP), resulting in increased disease risks in both developing and developed countries [2]. Diesel and petrol are the most commonly used vehicle fuels, which produce several toxic substances during combustion, such as benzene, formaldehyde, polycyclic aromatic hydrocarbons, nitrogen oxides, metals, and particulate matters (PMs). While the gaseous components dissipate rapidly, the solid components, PMs, can stay longer in the air. Moreover, there is a large surface area on PMs, especially PM2.5, allowing different kinds of carcinogens to be attached to the surface. These carcinogenic chemicals can exert adverse effects on human health, leading to cellular inflammatory responses and oxidative damage to impair lung function; cellular injuries can even result in the development of lung cancers [3–5]. There is a significant correlation between TRAP PM exposure and the incidence and mortality of lung cancers. Exposure to a high density of traffic, in particular PM2.5 and NO_2_ inside TRAP, was responsible for the increased risks and mortality of respiratory cancers, especially lung malignancies such as non-small cell lung cancer (NSCLC) adenocarcinoma and squamous cells carcinomas [6–8]. NSCLC is also the most common type of lung malignancy and accounts for about 85% of the total cases [9]. In addition, lung cancer patients with immunodeficiency are more susceptible to toxic substances compared to healthy individuals, leading to increased mortality [10]. Therefore, air pollution is an important trigger to the high incidence and mortality of lung cancers.

NSCLC includes lung squamous cell carcinoma (LUSC), lung adenocarcinoma (LUAD), and large-cell carcinoma (LCC), with LUAD and LUSC the most prominent subtypes [11]. Recently, although great efforts have been made in the technical improvement in early-diagnosis, surgery, and molecular targeted drugs, the prognosis of patients with NSCLC remains poor due to the absence of early symptoms leading to late diagnosis and metastasis [12–14]. Therefore, there is still a strong demand to identify novel and sensitive markers for early diagnosis and prognosis of patients with NSCLC.

Periostin (POSTN), an extracellular matrix (ECM) protein, is either absent or present at low levels in normal tissues but overexpressed in damaged, inflammatory and malignant tissues [15, 16]. POSTN is involved in embryonic development and tissue repair. POSTN normally interacts with other extracellular matrix proteins (such as tenascin-C, fibronectin, and type I collagen), enzymes (Bone morphogenetic protein-1 and lysyl oxidase), matricellular proteins (βig-h3 and cellular communication network factor 3), and receptors (integrins and Notch-1). Therefore, it plays a critical role in regulating cancer cell proliferation, invasion, angiogenesis, and metastasis [17, 18]. Furthermore, POSTN exerts a vital role in the communication between tumour cells and the surrounding microenvironments, thereby promoting the process of establish and remodel the tumour microenvironment (TME) [16, 19, 20]. Previous studies have also confirmed that high serum POSTN levels are associated with the prognosis of several cancers, such as breast cancer, ovarian cancer, colorectal cancer, and pancreatic cancer [15, 21–24]. POSTN is significantly downregulated in gastric cancer, and it can be used as a predictor of lymph node metastasis in such patients [25]. In lung cancer, the upregulation of POSTN correlates with the high proliferation and migration of human lung adenocarcinoma cell line (A549) [26]. Therefore, POSTN has a potential to be a novel marker of diagnosis and prognosis of lung cancer. However, the clinical significance of POSTN in lung cancer prognosis and correlation with immune infiltrate is still unknown, which formed the rationale of this study.

Therefore, in this study, we used informatics analysis to investigate the correlation between POSTN levels and the prognosis of lung cancer using existing data from The Cancer Genome Atlas (TCGA). In addition, the noncoding RNA, microRNA (miRNA), was also investigated due to its role as a vital regulator of the transcriptome in both physiological and pathological processes. Taken together, our finding emphasises that POSTN is related to poor prognosis and tumour immune infiltration in lung cancer patients.

## MATERIALS AND METHODS

UCSC Xena database (http://xena.ucsc.edu/) contains multiple public databases, such as TCGA, GTEx, TARGET, ICGC [27]. Gene Expression Omnibus (GEO) database (https://www.ncbi.nlm.nih.gov/gds) is a public repository that stores high throughput gene expression data sets, as well as original series and platform records. We downloaded the RNA-seq profiles in both tumour tissues and normal lung tissues from patients with LUAD and LUSC from UCSC Xena database and GEO database.

### Oncomine database

The differential expression level of the POSTN gene in various cancers was analysed in Oncomine database (http://www.oncomine.org). The thresholds were set as *P* value = 0.001, fold change of 2, and top 10% among the gene ranking.

### Gene correlation analysis in GEPIA

The online database Gene Expression Profiling Interactive Analysis (GEPIA) (http://gepia.cancer-pku.cn/index.html) was used to further confirm the mRNA expression of POSTN in tissues from patients with LUAD and LUSC. Furthermore, the correlation between POSTN levels and the expression of CD274, PDCD1, and CTLA4 mRNA was also performed using GEPIA. The Spearman method was used to determine the correlation coefficient.

### UALCAN database

UALCAN (http://ualcan.path.uab.edu/) is an interactive web resource for analysing publicly available cancer data. We used this database to analyse the expression of POSTN in normal tissues and cancer tissues from lung cancer patients based on cancer stages, smoking habits, gender, and node metastasis status.

### Evaluation of immune cell infiltration

TIMER (https://cistrome.shinyapps.io/timer/) was used to analyse the expression of POSTN in different types of cancer in the Diff Exp module, and the correlation between POSTN and immune cell infiltration, including B cells, CD8+ T cells, neutrophils, CD4+ T cells, macrophages, and dendritic cells via the gene module Furthermore, the correlation between POSTN and the expression of immune cell markers was analysed through a correlation module. The Spearman method was used to determine the correlation coefficient.

Genomic expression data from the TCGA were divided into high and low expression groups by the expression level of POSTN. The immune infiltration scores of 22 immune cell subtypes were assessed by the CIBERSORT package based on POSTN expression.

### Kaplan-Meier plotter database

Kaplan-Meier plotter (http://kmplot.com/analysis/) is an online platform for investigating the correlation between gene expression and survival rate. It contains more than 54,000 genes and survival data from 10,461 cancer samples [27]. The correlation between POSTN expression and survival rate (overall survival (OS), progress-free survival (FPS), and post-progression survival (PPS)) in patients with lung cancers was performed by Kaplan-Meier plotter. The hazard ratio (HR) with 95% confidence intervals and log-rank *P* value were determined.

### Gene interaction network construction

The STRING database (http://www.string-db.org/) is a public data to analyse the protein-protein interactions (PPI). The GeneMANIA (http://www.genemania.org) database was used to predict the genes related to the targeted genes. Here STRING and GeneMANIA were used to predict the genes and proteins interacting with POSTN.

### TISIDB database analysis

Using the TISIDB database, the interactions between tumours and the immune system can be detailed investigated. Nine hundred and eighty-eight immune-related anti-tumour genes from seven public databases can be interrogated in TISIDB, which contains different immune cell data, such as lymphocytes, immunomodulators, and chemokines. Thus, TISIDB was used to identify the correlations between POSTN expression and lymphocytes, chemokines, and immunomodulators.

### The human protein atlas

The Human Protein Atlas (http://www.proteinatlas.org) is an international program, containing the annotation of sixteen million tissue images among normal and cancer tissues. In this study, we used the Human Protein Atlas database to compare the protein expression of POSTN in normal lung tissue and lung squamous cell carcinoma tissue.

### Linkedomics database analysis

LinkedOmics (http://www.linkedomics.org/) is a public platform containing comprehensive cancer-associated multi-dimensional datasets among 32 TCGA cancer types. POSTN co-expression was analysed by Pearson test in LUAD and LUSC cohorts, presented in volcano plots, heat maps, or scatter plots. The top 50 genes positively and inversely related to POSTN in LUAD and LUSC were identified by LinkedOmics. Furthermore, Gene Ontology biological process (GO_BP) and Kyoto Encyclopedia of Genes and Genomes (KEGG) pathways were conducted through Gene Set Enrichment Analysis (GSEA) in the linkInterpreter module. The rank criterion was false discovery rate (FDR) <0.05 and 1000 simulations were performed.

### miRNA regulation of POSTN

Multiple target gene prediction tools, including miRWalK, miRANDA, TargetScan, and miRmap were used to predict the POSTN-targeted miRNAs. The correlation analysis for miRNA and POSTN co-expression were additionally analysed using starBase (http://starbase.sysu.edu.cn/). In addition, the survival analysis and co-expression of miRNA in LUSC and normal controls were also performed using starBase. How cancer prognosis was affected by POSTN-targeted miRNAs was investigated using miRNACancerMAP database.

### Statistical analysis

The statistical analyses were performed with R (V 4.1.1). Receiver operating characteristic (ROC) curve was also conducted for risk scores of POSTN using the pROC package. Univariate and multivariate Cox regression models were employed to analyse the prognostic value of POSTN in patients with LUAD and LUSC. The Chi-square test or Fisher’s exact test were used to analyse the relationship between POSTN and clinicopathological characteristics. The POSTN expression was analysed by the Oncomine, TIMER, GEPIA, UALCAN, GEO, and TCGA databases. Kaplan-Meier plotter database was used to perform survival curves. The Spearman method was applied to determine the correlation coefficient in the TIMER and GEPIA, while the Pearson method was performed in the LinkedOmics. *P* < 0.05 or log-rank *p* < 0.05 was considered as statistically significant.

## RESULTS

### Expression of POSTN in patients with lung cancer

**Figure 1:**
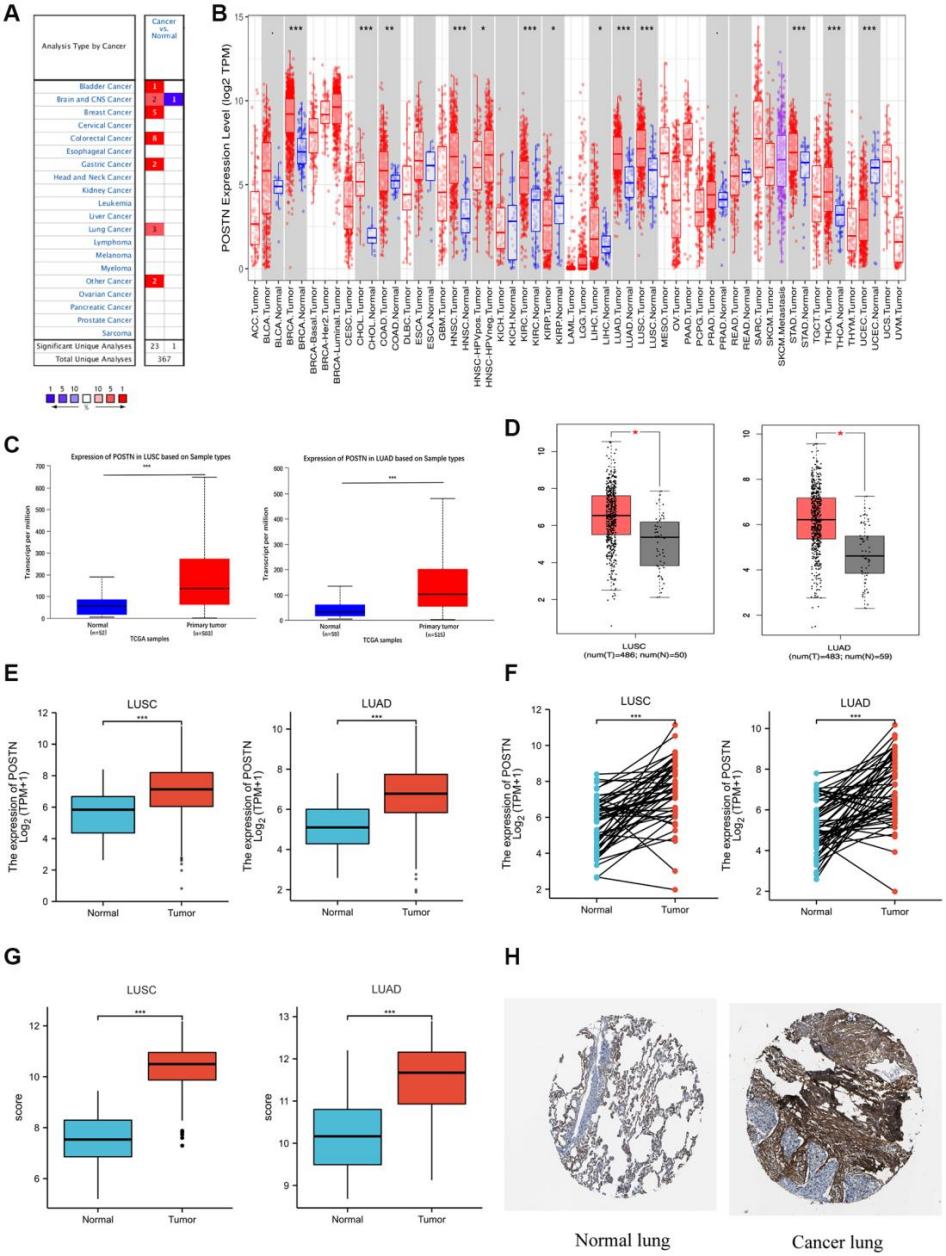
**Expression of POSTN in lung cancer.** POSTN expression in different cancers tissues compared to normal tissues based on the Oncomine database (**A**). POSTN expression in different tumour types based on the TIMER database (**B**). mRNA level of POSTN in lung cancer determined by UALCAN (**C**), GEPIA (**D**), and TCGA (**E**) databases. POSTN expression in 57 pairs of LUAD tissues and adjacent healthy tissues and 49 pairs of LUSC tissues and adjacent healthy tissues using TCGA database (**F**). POSTN expression in LUSC and LUAD based on the GEO database (**G**). The protein levels of POSTN based on the Human Protein Atlas (**H**). ^*^p < 0.05, ^***^p < 0.001.

We investigated the mRNA expression of POSTN among multiple human cancer types using the Oncomine database and Timer database (Figure 1A and 1B). Compared to healthy tissues, the mRNA level of POSTN was significantly upregulated in most cancer types, especially bladder urothelial carcinoma (BLCA), breast invasive carcinoma (BRCA), cholangiocarcinoma (CHOL), head and neck squamous cell carcinoma (HNSC), kidney renal clear cell carcinoma (KIRC), LUSC, LUAD, stomach adenocarcinoma (STAD), and thyroid carcinoma (THCA). In addition, POSTN expression was also higher in large cell lung carcinoma compared to healthy individuals (Supplementary Table 1 and Supplementary Figure 1). Furthermore, we found that POSTN was upregulated in LUAD and LUSC tissue using GEPIA and UALCAN database, consistent with the data in the TCGA database (Figure 1C–1E). These results were consistent in 57 and 49 pairs of tumour samples and adjacent normal samples in LUAD and LUSC, respectively (Figure 1F). As shown in Figure 1G, GSE10072 dataset was used to analyse the difference in POSTN expression between LUAD and normal tissue. We compared POSTN expression levels between LUSC and normal tissues using GSE44077. Similarly, POSTN was upregulated in LUAD and LUSC tissues based on GEO database. As shown in Figure 1H, immunohistochemical staining results from the Human Protein Atlas also demonstrated increased protein levels of POSTN in LUAD and LUSC tissues compared with adjacent healthy lung tissue. Therefore, both mRNA expression and protein level of POSTN are increased in lung cancer tissues.

### Correlations between POSTN expression and pathology

**Figure 2:**
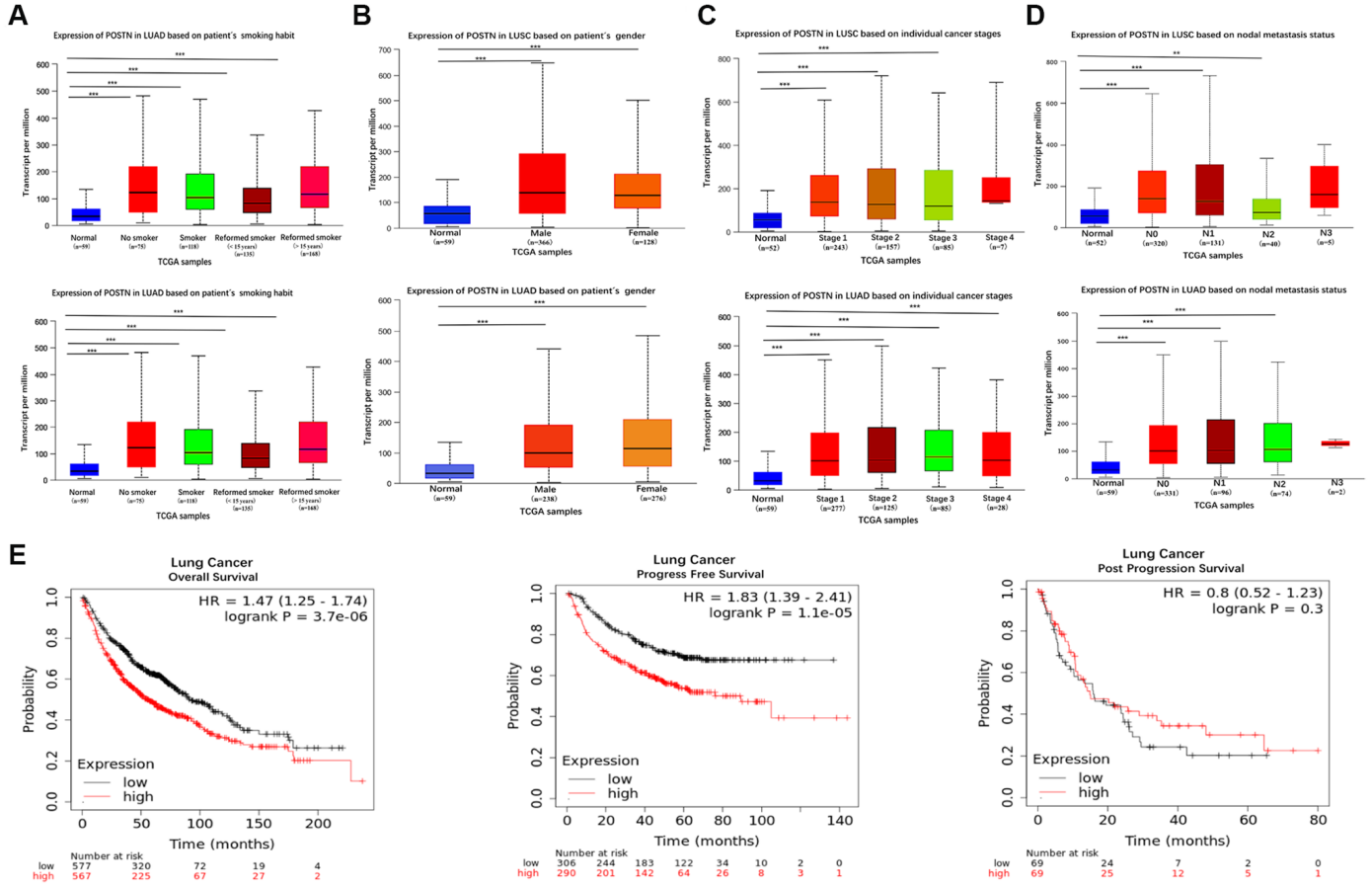
**Box plots evaluating POSTN mRNA expression based on clinical parameters and the prognosis in lung cancer.** POSTN mRNA expressions were remarkably correlated with LUAD and LUSC patients’ individual cancer smoking habits (**A**), gender (**B**), status (**C**), nodal metastasis (**D**). Survival curves using the Kaplan-Meier plotter are shown for overall survival, progression-free survival, and progression-free survival (**E**). ^**^p < 0.01, ^***^p < 0.001.

To reveal the role of POSTN in LUAD and LUSC progression, the correlation between POSTN expression and risk factors, as well as pathological parameters, were performed using the UALCAN database, including smoking habits, gender, individual cancer stages, and lymph node metastasis status in lung cancer patients. POSTN was significantly upregulated in lung cancer patients who smoked compared to healthy controls (Figure 2A), with no sex differences (Figure 2B). The mRNA levels of POSTN in LUSC with stages 1, 2, and 3 and in LUAD with stages 1, 2, 3, and 4 were higher than that in healthy controls (Figure 2C). POSTN was also found to be increasingly expressed in lymph node metastasis, especially in N0 and N1 in LUSC and N0, N1, and N2 in LUAD (Figure 2D). Similarly, as shown in Table 1, POSTN expression correlates to cancer pathologic stages (*P* = 0.048), N stage (*P* = 0.024), M stage (*P* = 0.015), and age (*P* = 0.037); while no significant correlation was found with T stage, gender, race and smoking status of the LUSC patients. However, there was no correlation between POSTN expression with clinicopathologic characteristics among patients with LUAD. The findings indicated that the overexpression of POSTN has a vital role in the progress of lung cancer and metastasis.

**Table 1:** Correlation between POSTN and clinicopathologic characteristics.

**Characteristic**	**LUSC**			**LUAD**		
	**POSTN expression**		***p***	**POSTN expression**		***p***
	**Low**	**High**		**Low**	**High**	
***n***	250	251		256	257	
**T stage**			0.410			0.061
T1	64 (12.8%)	50 (10%)		97 (19%)	71 (13.9%)	
T2	144 (28.7%)	149 (29.7%)		131 (25.7%)	145 (28.4%)	
T3	32 (6.4%)	39 (7.8%)		18 (3.5%)	29 (5.7%)	
T4	10 (2%)	13 (2.6%)		9 (1.8%)	10 (2%)	
**N stage**			0.024			0.265
N0	154 (31.1%)	165 (33.3%)		173 (34.5%)	157 (31.3%)	
N1	63 (12.7%)	68 (13.7%)		43 (8.6%)	52 (10.4%)	
N2	29 (5.9%)	11 (2.2%)		34 (6.8%)	40 (8%)	
N3	2 (0.4%)	3 (0.6%)		0 (0%)	2 (0.4%)	
**M stage**			0.015			0.569
M0	205 (49%)	206 (49.3%)		165 (44.7%)	179 (48.5%)	
M1	0 (0%)	7 (1.7%)		14 (3.8%)	11 (3%)	
**Pathologic stage**			0.048			0.147
I	121 (24.3%)	123 (24.7%)		149 (29.5%)	125 (24.8%)	
II	83 (16.7%)	79 (15.9%)		54 (10.7%)	67 (13.3%)	
III	45 (9.1%)	39 (7.8%)		36 (7.1%)	48 (9.5%)	
IV	0 (0%)	7 (1.4%)		14 (2.8%)	12 (2.4%)	
**Gender**			0.345			0.567
Female	70 (14%)	60 (12%)		134 (26.1%)	142 (27.7%)	
Male	180 (35.9%)	191 (38.1%)		122 (23.8%)	115 (22.4%)	
**Race**			0.623			0.210
Asian	4 (1%)	5 (1.3%)		6 (1.3%)	1 (0.2%)	
Black or African American	18 (4.6%)	12 (3.1%)		26 (5.8%)	26 (5.8%)	
White	179 (46.1%)	170 (43.8%)		193 (43.3%)	194 (43.5%)	
**Age**			0.037			0.726
≤65	106 (21.5%)	84 (17.1%)		120 (24.3%)	118 (23.9%)	
>65	138 (28%)	164 (33.3%)		124 (25.1%)	132 (26.7%)	
**Smoker**			0.776			0.348
No	10 (2%)	8 (1.6%)		33 (6.6%)	41 (8.2%)	
Yes	232 (47.4%)	239 (48.9%)		218 (43.7%)	207 (41.5%)	
**OS**			0.064			0.484
Alive	153 (30.5%)	132 (26.3%)		167 (32.6%)	159 (31%)	
Dead	97 (19.4%)	119 (23.8%)		89 (17.3%)	98 (19.1%)	
**DSS**			0.524			0.499
Alive	186 (41.4%)	174 (38.8%)		182 (38.2%)	180 (37.7%)	
Dead	42 (9.4%)	47 (10.5%)		53 (11.1%)	62 (13%)	
**PFI**			0.576			0.389
Alive	180 (35.9%)	174 (34.7%)		157 (30.6%)	147 (28.7%)	
Dead	70 (14%)	77 (15.4%)		99 (19.3%)	110 (21.4%)	

### Prognostic value and POSTN mRNA expression in lung cancer patients

To evaluate the role of POSTN in cancer prognosis, we examined the correlation between POSTN expression and overall survival, progression-free survival, and post-progression survival using the Kaplan-Meier plotter database (Figure 2E). The results showed that patients with overexpressed POSTN had poorer overall survival and progression-free survival than those with low POSTN expression (both log-rank *P* < 0.001). In univariate analysis, as shown in Tables 2 and 3, T stage (HR 1.658; 95% CI 1.200, 2.291; *P* = 0.002), M stage (HR 3.112; 95% CI 1.272, 5.664; *P* = 0.013), and pathologic stage (HR 1.570; 95% CI 1.139, 2.163; *P* = 0.006) were associated with OS in patients with LUSC; whereas T stage (HR 2.317; 95% CI 1.591, 3.375; *P* < 0.001), N stage (HR 2.321; 95% CI 1.631, 3.303; *P* < 0.001), M stage (HR 2.136; 95% CI 1.248,3.653; *P* = 0.006), and pathologic stage (HR 2.664; 95% CI 1.960,3.621; *P* < 0.001) were associated with OS in patients with LUAD. In multivariate analysis, only age (for OS, HR 0.688; 95% CI 0.500, 0.948; *P* = 0.022) was responsible for the OS of LUSC patients; while only T stage (for OS, HR 1.70; 95% CI 1.063, 2.730; *P* = 0.027) was independent prognostic factors for LUAD. Furthermore, clinicopathologic characteristics (including TNM stage, pathologic stage, gender, age, as well as smoking) were included to build a prognostic nomogram based on TCGA database, which can be used to predict the survival probabilities of OS at 1-, 3-, and 5-years for patients with LUSC and LUAD. Collectively, these results indicate that overexpression of POSTN may be an independent prognostic factor for lung cancer patients.

**Table 2:** Univariate and multivariate Cox proportional hazards analysis of POSTN expression and OS for patients with LUSC.

**Characteristics**	**Total (*N*)**	**Univariate analysis**		**Multivariate analysis**	
		**Hazard ratio (95% CI)**	***P* value**	**Hazard ratio (95% CI)**	***P* value**
**T stage**	496	1.658 (1.200–2.291)	**0.002**	1.325 (0.844–2.078)	0.221
**N stage**	490	1.354 (0.877–2.090)	0.171		
**M stage**	415	3.112 (1.272–7.616)	**0.013**	2.509 (0.963–6.537)	0.060
**Pathologic stage**	492	1.570 (1.139–2.163)	**0.006**	1.199 (0.758–1.896)	0.437
**Gender**	496	1.211 (0.879–1.669)	0.241		
**Smoker**	484	1.708 (0.754–3.868)	0.199		
**Age**	490	0.782 (0.587–1.042)	0.093	0.688 (0.500–0.948)	**0.022**

**Table 3:** Univariate and multivariate Cox proportional hazards analysis of POSTN expression and OS for patients with LUAD.

**Characteristics**	**Total (*N*)**	**Univariate analysis**		**Multivariate analysis**	
		**Hazard ratio (95% CI)**	***P* value**	**Hazard ratio (95% CI)**	***P* value**
**T stage**	523	2.317 (1.591–3.375)	**<0.001**	1.704 (1.063–2.730)	**0.027**
**N stage**	510	2.321 (1.631–3.303)	**<0.001**	1.329 (0.633–2.788)	0.452
**M stage**	377	2.136 (1.248–3.653)	**0.006**	1.212 (0.540–2.720)	0.641
**Pathologic stage**	518	2.664 (1.960–3.621)	**<0.001**	1.848 (0.848–4.024)	0.122
**Gender**	526	1.070 (0.803–1.426)	0.642		
**Smoker**	512	1.119 (0.742–1.688)	0.591		
**Age**	516	0.817 (0.612–1.092)	0.172		

### Regulatory network of POSTN in lung cancer

**Figure 3:**
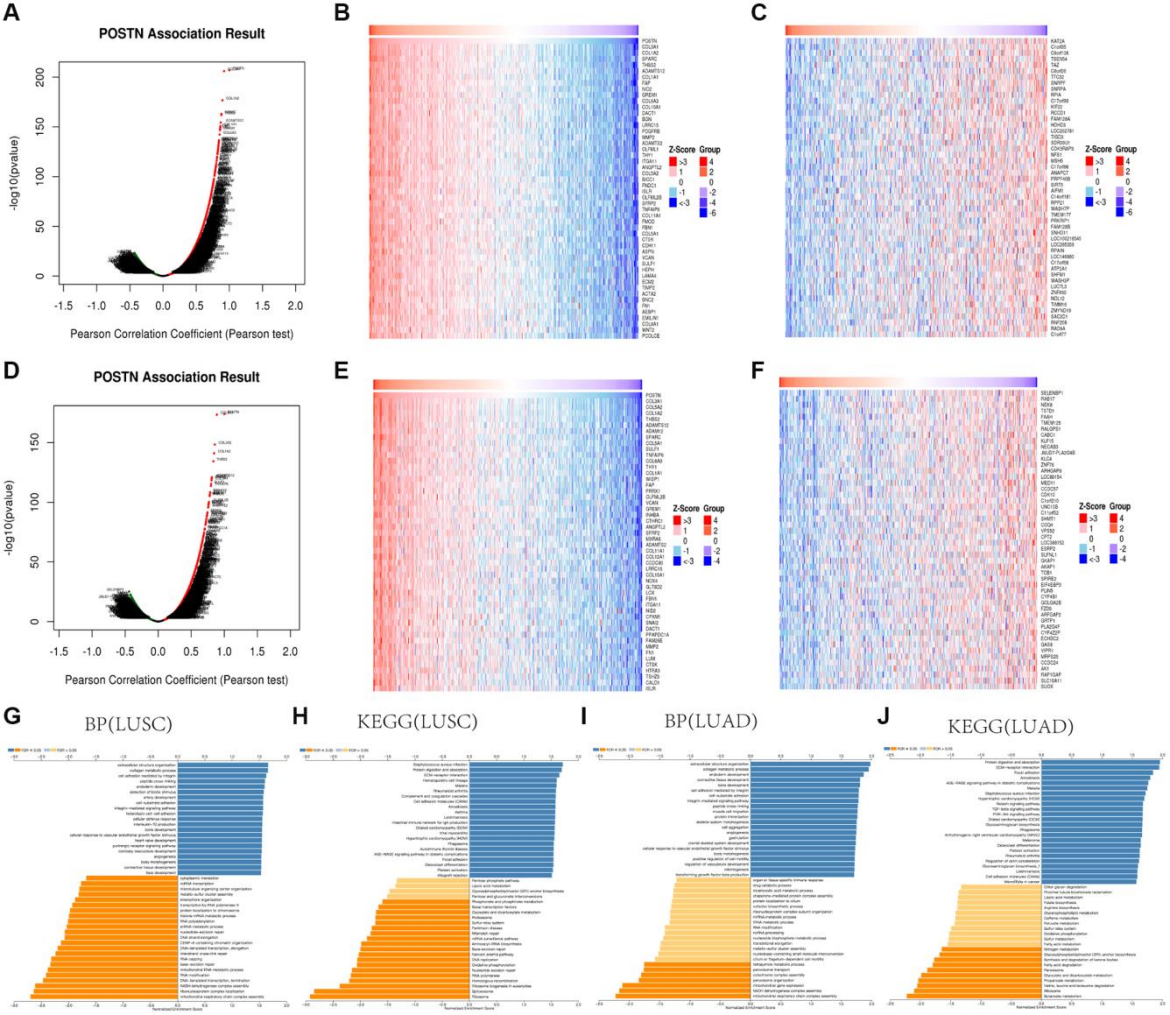
**GO and KEGG enrichment analysis for POSTN in LUAD and LUSC.** Volcano plot showing the correlations between POSTN and genes differentially expressed in LUAD (**A**). Heat maps showing genes positively and negatively correlated with POSTN in LUAD (Top50) (**B**, **C**). Volcano plot showing the correlations between POSTN and genes differentially expressed in LUSC (**D**). Heat maps showing genes positively and negatively related with POSTN in LUSC (Top50) (**E**, **F**). Enrichment terms in BP and KEGG enrichment pathways in LUAD and LUSC (**G**–**J**).

To identify the biological implications of POSTN in lung cancer, we examined the genes that were correlated with POSTN in LUAD and LUSC using the LinkedOmics database. As shown in Figure 3A, 6156 genes (red dots) positively correlated with POSTN, and 5554 genes (green dots) were inversely correlated in LUSC (FDR <0.05, Figure 3A). Furthermore, there are 5875 genes positively and 5077 genes inversely correlated with POSTN in LUAD (FDR <0.05, Figure 3D). The heatmaps illustrated the top 50 genes with positive and negative correlations with POSTN in LUSC (Figure 3B and 3C) and LUAD (Figure 3E, 3F). These genes correlated to POSTN were mainly involved in the extracellular matrix structure, collagen metabolism, cell adhesion, and integrin-mediated signalling pathway (Figure 3G, 3I). KEGG analysis showed enrichment of some pathways in lung cancer patients, such as protein digestion and absorption, ECM-receptor interaction, intestinal immune network for IgA production, cell adhesion molecules, relaxin signalling pathway, transforming growth factor-beta signalling pathway, platelet activation, and PI3K-Akt signalling pathway (Figure 3H, 3J), suggesting that the upregulation of POSTN is linked to the micro-environment and immune response to support cancer progression.

**Figure 4:**
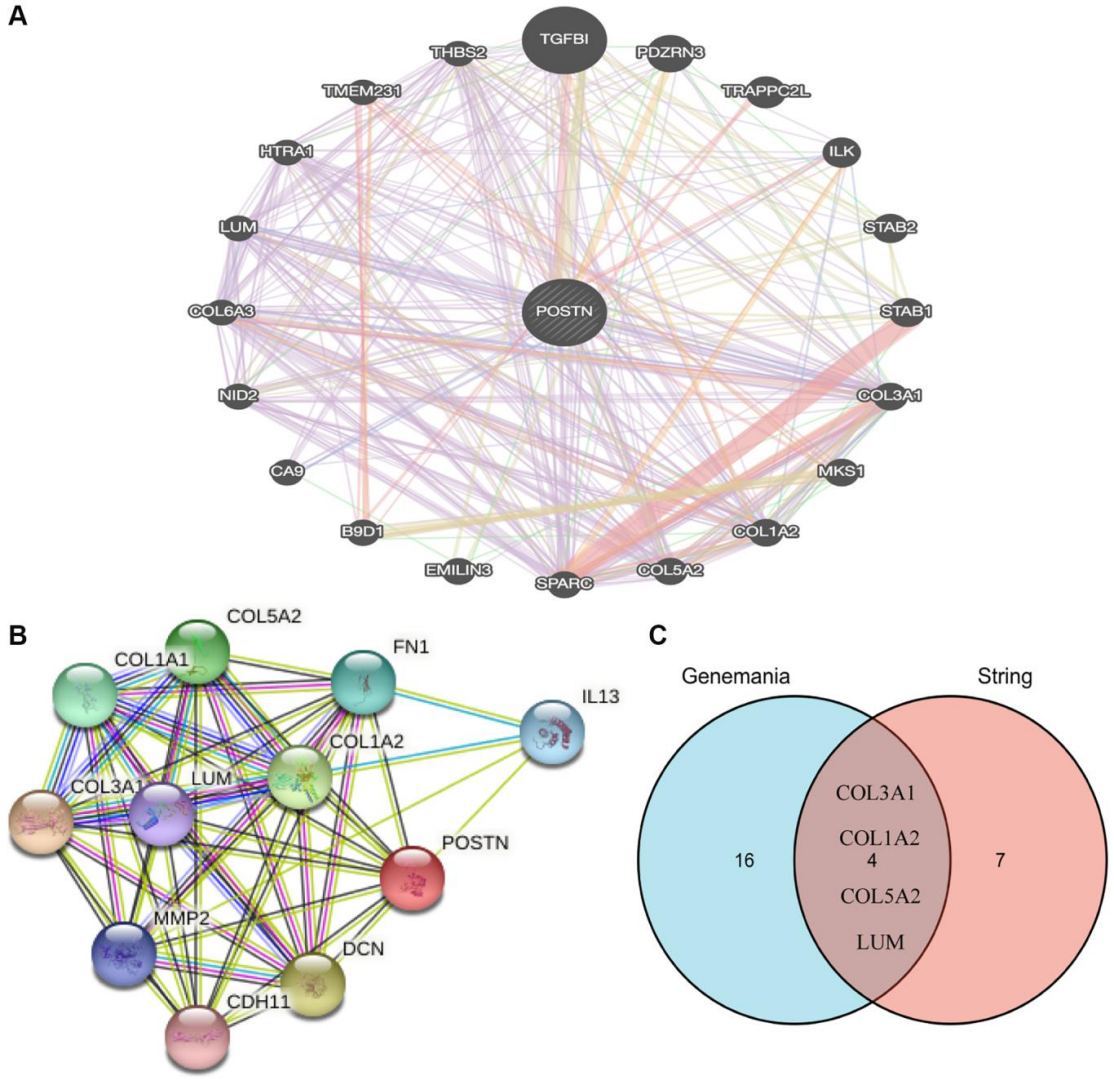
**Analysis of neighboring gene networks in lung cancer.** The gene-gene interaction network of POSTN constructed using GeneMania (**A**). The PPI network of POSTN generated using STRING (**B**). The overlapping genes (**C**).

To further investigate the function of POSTN, the STRING database and GeneMANIA database were used (Figure 4A, 4B). We identified the common gene hubs from these two databases: COL3A1, COL1A2, COL5A2, and LUM (Figure 4C), which were the top 50 genes positively correlated with POSTN in LUSC patients. All these common gene hubs are ECM related proteins, which could be involved in the process of cancer cell proliferation, invasion and metastasis, showing that POSTN could regulate the microenvironment of tumor and exerted a significant role in cancer development, especially in LUSC.

### Correlation between POSTN expression and immune cell infiltration in lung cancer

**Figure 5:**
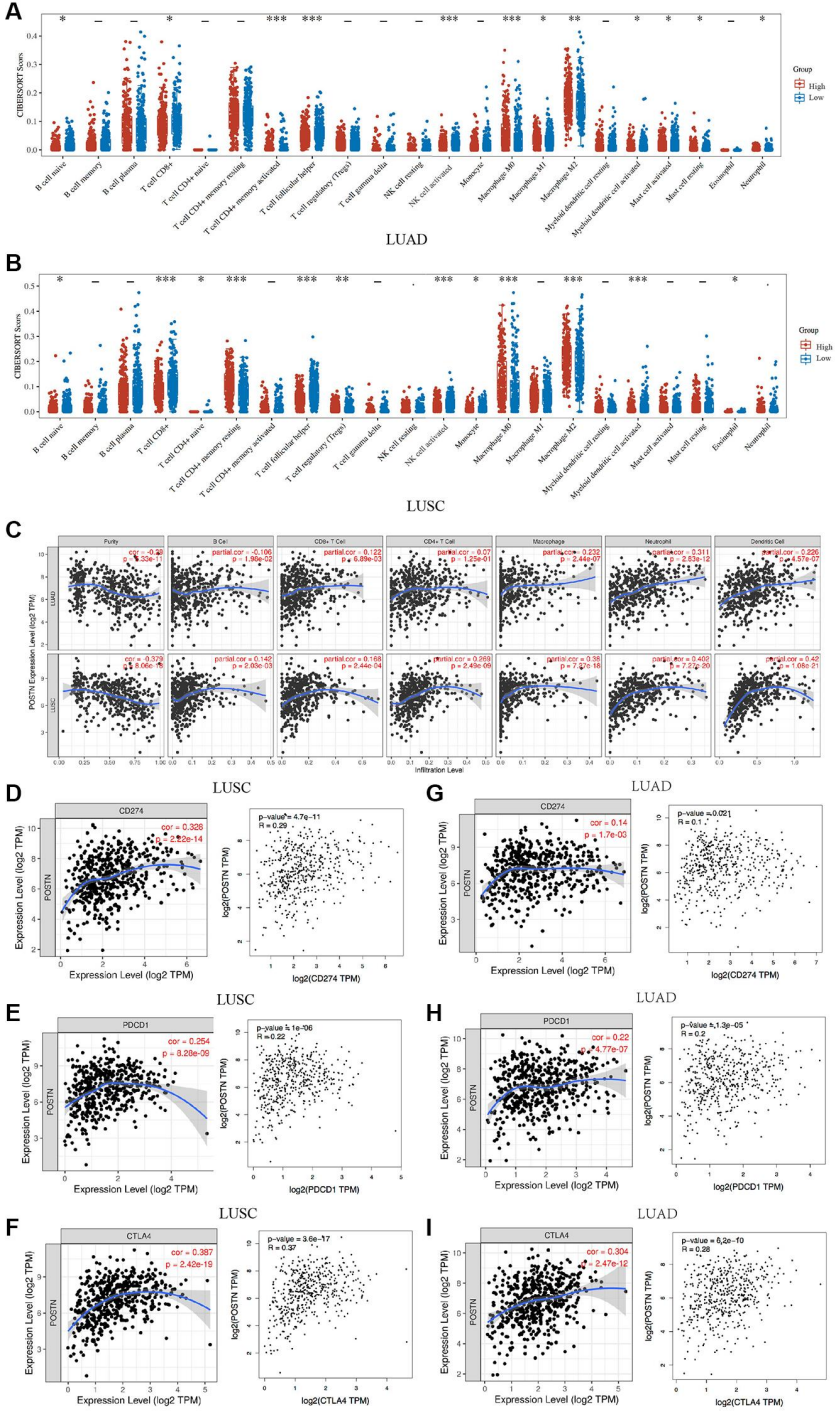
**Correlation between POSTN expression and immune infiltration.** The correlations between POSTN expression and immune cells infiltration in LUSC and LUAD (**A** and **B**). POSTN is significantly associated with cancer purity and positively correlates with the infiltration of different immune cells in LUAD and LUSC using the TIMER database (**C**). The correlations between POSTN expression and checkpoint in LUSC and LUAD, including CD274, PDCD1, and CTLA-4 (**D**–**I**). ^*^p < 0.05, ^**^p < 0.01, ^***^p < 0.001.

To investigate potential role of POSTN in immune cell infiltration, we performed an integrated analysis using TIMER database. As shown in Figure 5A–5C and Supplementary Figure 2, the immune cell infiltration was significantly different between tissues with overexpressed POSTN and those with downregulated POSTN from patients with LUAD and LUSC. POSTN expression was positively associated with the infiltration of immune cells in LUAD, including B cells (cor = 0.142, *P* = 2.03e-03), CD8+ T cells (cor = 0.168, *P* = 2.44e-04), CD4+T cells (cor = 0.269, *P* = 2.49e-09), macrophages (cor = 0.38, *P* = 7.27e-18), neutrophils (cor = 0.402, *P* = 7.27e-20), and dendritic cells (cor = 0.42, *P* = 1.08e-21). Similarly, the level of POSTN expression in LUSC tissues positively correlated with the infiltration levels of CD4+ T cells (cor = 0.007, *P* = 1.25e-01), CD8+ T cells (cor = 0.122, *P* = 6.89e-03), macrophages (cor = 0.232, *P* = 2.44e-07), neutrophils (cor = 0.311, *P* = 2.83e-12), and dendritic cells (cor = 0.226, *P* = 4.57e-07), while negatively correlated with B cells (cor = −0.106, *P* = 1.98e-02). These findings strongly suggest that POSTN plays an important role in immune infiltration in lung cancer, especially in LUSC.

### Correlation between POSTN expression and immune markers in lung cancer

We analysed correlations between POSTN and markers of various immune cells based on the TIMER databases. The expression of POSTN was significantly correlated with most markers of multiple immune cell subtypes in lung cancer (Table 4). Furthermore, the correlations between POSTN and various T cells were investigated to strengthen the understanding of the correlation between POSTN and immune response (Table 5). After adjustments for cancer purity in LUSC and LUAD, POSTN expression was strongly associated with the presence of B cells, CD8+ T cells, CD4+ T cells, neutrophils, dendritic cells, monocytes, and various functional T cells, including T helper, Tregs, resting Treg, effector Treg, naïve T cells, effector T cells, resistant memory, and exhausted T cells. In addition, T cell checkpoints, including CD274, PDCD1, and CTLA4, were assessed based on the Timer database and GEPIA database (Figure 5D–5I). POSTN is associated with immune cell infiltration and immune escape in the lung cancer microenvironment, especially LUSC.

**Table 4:** Correlation analysis between POSTN and markers of immune cells in TIMER.

**Immune cell**	**Markers**	**LUSC**				**LUAD**			
		**None**		**Purity**		**None**		**Purity**	
		**cor**	***p***	**cor**	***P***	**cor**	***P***	**cor**	***p***
**B cell**	CD19	0.273	^***^	0.103	^*^	0.143	^**^	−0.001	0.986
	CD79A	0.362	^***^	0.213	^***^	0.248	^***^	0.136	^**^
**CD8+ T cell**	CD8A	−0.269	^***^	−0.293	^***^	0.189	^***^	0.057	0.21
	CD8B	0.164	^***^	0.106	^**^	0.142	^**^	0.035	0.433
**CD4+ T cell**	CD4	0.554	^***^	0.458	^***^	0.341	^***^	0.241	^***^
**T cell (general)**	CD2	0.343	^***^	0.21	^***^	−0.242	^***^	0.334	0.446
	CD3E	0.353	^***^	0.214	^***^	0.185	^***^	0.021	0.688
	CD3D	0.292	^***^	0.15	^**^	0.204	^***^	0.054	0.28
**M1**	IRF5	0.036	0.425	^**^	0.847	0.17	^***^	0.084	0.0608
	PTGS2	0.225	^***^	0.187	^***^	0.246	^***^	0.255	^***^
	NOS2	0.032	0.474	^*^	0.489	0.272	^***^	0.222	^***^
**M2**	CD163	0.522	^***^	0.446	^***^	0.357	^***^	0.292	^***^
	VSIG4	0.481	^***^	0.397	^***^	0.274	^***^	0.214	^***^
	MS4A4A	0.491	^***^	0.4	^***^	0.31	^***^	0.246	^***^
**Neutrophils**	CEACAM8	0.06	0.182	^*^	0.554	−0.104	^*^	−0.117	^**^
	ITGAM	0.502	^***^	0.408	^***^	0.294	^***^	0.216	^***^
	CCR7	0.302	^***^	0.156	^***^	0.138	^**^	−0.016	0.721
**Dendritic cell**	HLA-DPA1	0.419	^***^	0.267	^***^	0.062	0.161	−0.054	0.232
	HLA-DPB1	0.424	^***^	0.261	^***^	0.025	0.575	−0.105	^*^
	HLA-DQB1	0.341	^***^	0.241	^***^	0.013	0.761	−0.102	^*^
	HLA-DRA	0.417	^***^	0.304	^***^	0.088	^*^	−0.029	0.517
	CD1C	0.288	^***^	0.108	^*^	−0.01	0.829	−0.096	^*^
	NRP1	0.555	^***^	0.486	^***^	0.317	^***^	0.3	^***^
	ITGAX	0.45	^***^	0.313	^***^	0.221	^***^	0.099	^*^
**Natural killer cell**	KIR2DL1	0.073	0.104	0.01	0.831	0.049	0.266	0.004	0.931
	KIR2DL3	0.053	0.235	−0.011	0.814	0.142	^**^	0.076	0.091
	KIR2DL4	0.031	0.486	−0.056	0.222	0.212	^***^	0.139	^**^
	KIR3DL1	0.131	^***^	0.047	0.302	0.024	0.582	−0.032	0.475
	KIR3DL2	0.058	0.197	−0.037	0.418	0.159	^***^	0.094	^*^
	KIR3DL3	−0.044	0.329	−0.069	0.132	0.102	^*^	0.084	0.0628
	KIR2DS4	0.106	^***^	0.051	0.263	0.093	^*^	0.04	0.378
**Monocyte**	CD86	0.486	^***^	0.375	^***^	0.388	^***^	0.304	^***^
	CSF1R	0.565	^***^	0.474	^***^	0.359	^***^	0.278	^***^
**TAM**	CCL2	0.561	^***^	0.501	^***^	0.321	^***^	0.248	^***^
	CD68	0.457	^***^	0.36	^***^	0.254	^***^	0.174	^***^
	IL10	0.468	^***^	0.402	^***^	0.271	^***^	0.18	^***^

^*^p < 0.05, ^**^p < 0.01, ^***^p < 0.001.

**Table 5:** Correlation analysis between POSTN and markers of different T cell types in TIMER.

**Description**	**Markers**	**LUSC**				**LUAD**			
		**None**		**Purity**		**None**		**Purity**	
		**cor**	***p***	**cor**	***P***	**cor**	***P***	**cor**	***p***
**Th1**	TBX21	0.249	^***^	0.115	^*^	0.12	^***^	−0.026	0.566
	STAT4	0.398	^***^	0.279	^***^	0.173	^***^	0.035	0.442
	STAT1	0.217	^***^	0.157	^***^	0.361	^***^	0.232	^***^
	TNF	0.348	^***^	0.254	^***^	0.204	^***^	0.114	^*^
	IFNG	0.086	0.0548	0.004	0.0923	0.186	^***^	0.088	0.0507
**Th1-like**	HAVCR2	0.474	^***^	0.37	^***^	0.361	^***^	0.272	^***^
	IFNG	0.086	0.0548	0.004	0.923	0.186	^***^	0.088	0.0507
	CXCR3	0.334	^***^	0.204	^***^	0.155	^***^	0.023	0.612
	BHLHE40	0.203	^***^	0.206	^***^	0.244	^***^	0.231	^***^
	CD4	0.554	^***^	0.458	^***^	0.341	^***^	0.241	^***^
**Th2**	STAT6	−0.021	0.637	−0.026	0.576	−0.151	^**^	−0.147	^**^
	STAT5A	0.329	^***^	0.221	^***^	0.228	^***^	0.18	^**^
	FOXP3	0.521	^***^	0.419	^***^	0.371	^***^	0.269	^***^
	CCR8	0.548	^***^	0.459	^***^	0.393	^***^	0.314	^***^
	TGFB1	0.462	^***^	0.362	^***^	0.343	^***^	0.263	^***^
**Resting Tregs**	FOXP3	0.521	^***^	0.419	^***^	0.371	^***^	0.269	^***^
	IL2RA	0.505	^***^	0.422	^***^	0.47	^***^	0.411	^***^
**Effector Tregs**	FOXP3	0.521	^***^	0.419	^***^	0.371	^***^	0.69	^***^
	CCR8	0.548	^***^	0.459	^***^	0.393	^***^	0.314	^***^
**Effector T cells**	CX3CR1	0.378	^***^	0.285	^***^	−0.02	0.657	−0.088	0.0519
	FGFBP2	−0.021	0.643	0.015	0.764	−0.114	^**^	−0.169	^***^
	FCGR3A	0.527	^***^	0.452	^***^	0.432	^***^	0.364	^***^
**Naïve T cells**	CCR7	0.302	^***^	−0.414	^***^	0.138	^**^	−0.016	0.721
	SELL	0.321	^***^	0.157	^***^	0.236	^***^	0.09	^*^
**Effector memory T cells**	DUSP4	0.216	^***^	0.157	^***^	0.101	^*^	0.099	^*^
	GZMK	0.364	^***^	0.235	^***^	0.184	^***^	0.049	0.28
	GZMA	0.214	^***^	0.106	^*^	0.183	^***^	0.051	0.268
**Resistant memory T cells**	CD69	0.386	^***^	0.264	^***^	0.144	^**^	0.027	0.557
	CXCR6	0.319	^***^	0.2	^***^	0.208	^***^	0.071	0.114
	MYADM	0.553	^***^	0.507	^***^	0.382	^***^	0.316	^***^
**Exhausted T cells**	HAVCR2	0.474	^***^	0.37	^***^	0.361	^***^	0.272	^***^
	LAG3	0.184	^***^	0.084	0.062	0.203	^***^	0.085	0.0995
	CXCL13	0.319	^***^	0.204	^***^	0.187	^***^	0.048	0.287
	LAYN	0.211	^***^	0.205	^***^	0.499	^***^	0.435	^***^
**General memory T-cell**	CCR7	0.302	^***^	0.156	^***^	0.138	^**^	−0.016	0.721
	SELL	0.321	^***^	0.157	^***^	0.236	^***^	0.09	^*^
	IL7R	0.647	^***^	0.581	^***^	0.37	^***^	0.286	^***^

^*^p < 0.05, ^**^p < 0.01, ^***^p < 0.001.

**Figure 6:**
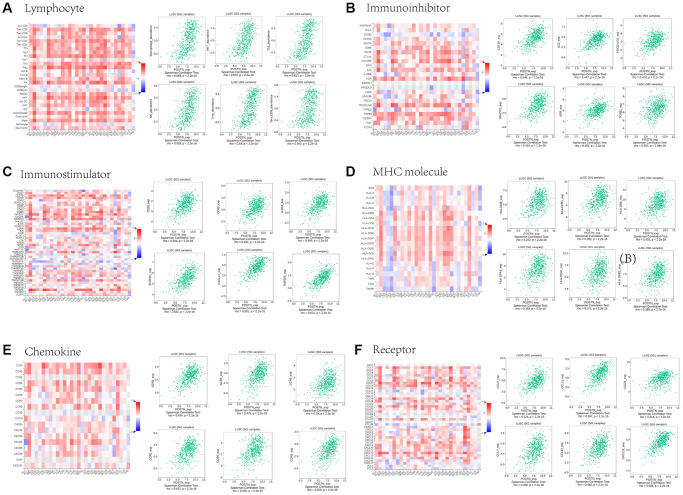
**Associations between POSTN expression and lymphocytes, immunomodulators, and chemokines in LUSC.** Correlations between tumour-infiltrating lymphocytes (TILs) and POSTN and the six TILs with the highest correlation values (**A**). Correlations between immunomodulators and POSTN and the six immunomodulators with the highest correlation values (**B**–**D**). Correlations between chemokines (or receptors) and POSTN and the six chemokines (or receptors) with the highest correlation values (**E** and **F**).

To further evaluate the role of POSTN in TME in LUSC, we used the TISIDB database to explore the relationship between POSTN and several immune molecules. We evaluated the correlation between the expression of POSTN and 28 tumour immune infiltrating cell subtypes (Figure 6A). Results showed that POSTN expression was positively correlated with macrophages (rho = 0.569, *P* < 2.2e-16), NK cells (rho = 0.583, *P* < 2.2e-16), NK T cells (rho = 0.572, *p* < 2.2e-16), Tem_CD8 cells (rho = 0.403, *p* < 2.2e-16), Th1 cells (rho = 0.612, *p* < 2.2e-16), and Treg cells (rho = 0.640, *p* < 2.2e-16). Furthermore, the correlations between POSTN expression and three types of immunomodulators were conducted, including immunoinhibitors, immunostimulators, and major histocompatibility complex molecules. Correlations between immunoinhibitors, including CSF1R, HAVCR2, IL10, KDR, PDCD1LG2, and TGFB1, and POSTN are shown in Figure 6B. Correlations between immunostimulators, including CD28, CD80, CXCL12, ENTPD1, IL2RA, and TNFSF4, and POSTN are shown in Figure 6C. Correlations between major histocompatibility complex molecules, including HLA-DMP, HLA-DPA1, HLA-DPB1, HLA-DQA1, HLA-DRA, and HLA-DRB1, with POSTN are shown in Figure 6D. Finally, the potential associations of POSTN with chemokines and chemokine receptors in LUSC were investigated, and the results demonstrated associations of POSTN with CCL2, CCL7, CCL11, CCL13, CCL21, CXCL12, CXCL10, CXCL13, CXCR1, CXCR2, CXCR4, CXCR5, CCR6, and CCR8 (Figure 6E and 6F). The results showed that POSTN may regulate immune molecules in the tumour microenvironment of LUSC via various pathways, thereby exerting a specific role in immune cell infiltration.

### Prognosis and POSTN expression in LUSC patients

**Figure 7:**
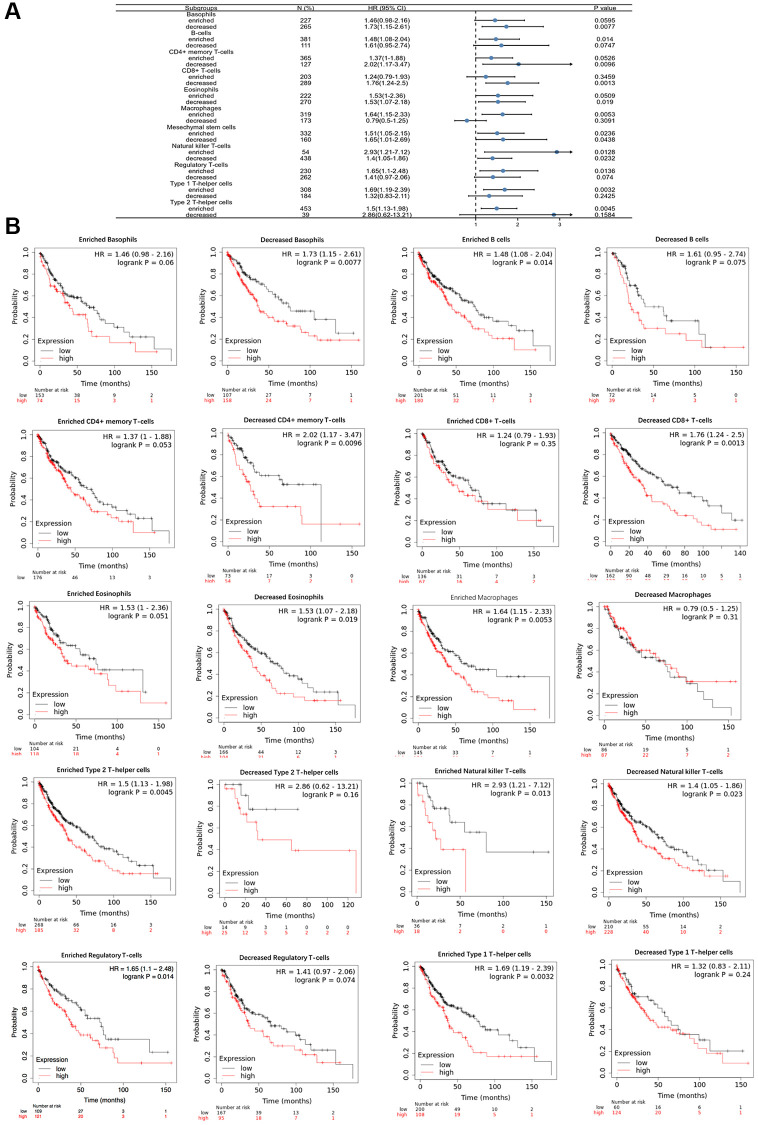
**Comparison of Kaplan-Meier survival curves of the high and low expression of POSTN in immune cell subgroups in LUSC.** A forest plot shows the prognostic value of POSTN expression based on different immune cell subgroups in LUSC (**A**). Correlations between the expression of POSTN and OS based on different immune cell subgroups in LUSC (**B**).

The above results suggest that POSTN is related to immune infiltration in LUSC patients. To assess the prognostic values of POSTN, we further investigated the correlation between immune infiltration and the prognosis of LUSC patients. As shown in Figure 7A and 7B, various immune cell subgroups were analysed in LUSC. Overexpression of POSTN in LUSC patients with decreased numbers of basophils, eosinophils, CD4+ memory T cells, and CD8+ memory T cells, as well as increased numbers of B cells, T helper cells, and Treg cells, had a poor prognosis. These results showed that high-level expression of POSTN may affect the prognosis of LUSC patients through immune infiltration.

### Associations between POSTN expression and target-microRNA

**Figure 8:**
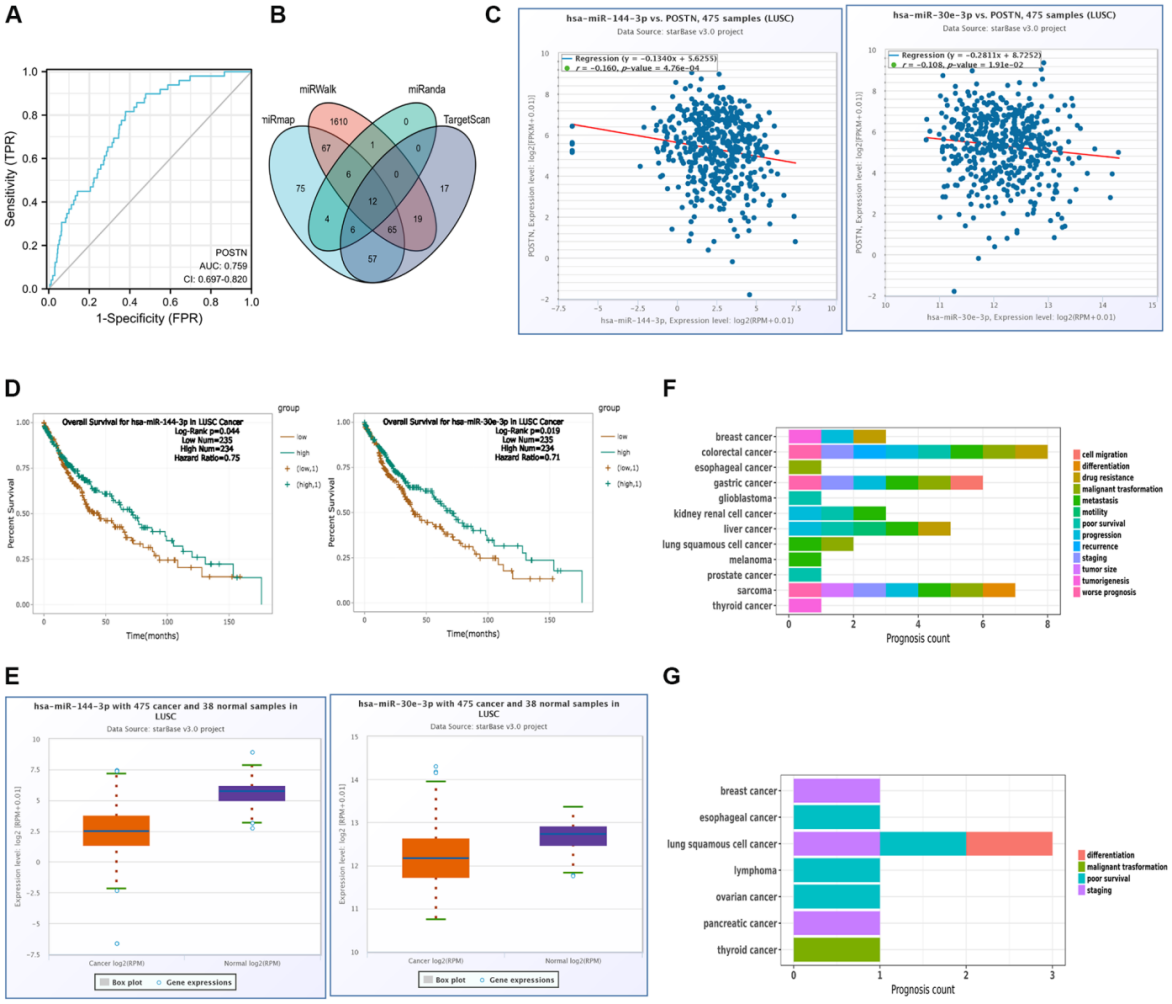
**miRNA regulation and diagnostic value of POSTN.** (**A**) ROC curve analysis of POSTN in LUSC. (**B**) The overlapping POSTN-targeted miRNAs. (**C**) The correlation between the expression of POSTN and POSTN-targeted miRNAs in LUSC and control normal samples determined by the starBase database. (**D**) The prognostic value of POSTN-targeted miRNAs in LUSC assessed by Kaplan-Meier plotter (**E**) The expression of POSTN-targeted miRNAs in LUSC and control normal samples determined by starBase database. (**F**, **G**) Prognosis affected by POSTN-targeted miRNAs was conducted using miRNACancerMAP database.

As shown in Figure 8A, we conducted a ROC curve analysis to evaluate the prognostic values of POSTN. The ROC curve analysis presented an AUC of 0.759 (95% CI, 0.697−0.820). At a cutoff of 5.096, POSTN had a sensitivity of 81.6.0% and a specificity of 62.2%. Therefore, POSTN can be a novel molecular of progression and development of LUSC.

To further investigate the role of POSTN in LUSC, target microRNAs that potentially bind to POSTN was predicted using the various databases. As shown in Figure 8B, there were 12 common miRNAs, which could potentially regulate the expression of POSTN. However, there were only 2 miRNAs, has-miR-144-3p and has-miR-30e-3p, significantly correlated to poor prognosis and were consistent with the principle of negative regulation of target genes (Figure 8C, 8D). As shown in Figure 8E, has-miR-144-3p and has-miR-30e-3p were markedly downregulated in LUSC compared to normal tissues and their expression was positively linked to the prognosis of LUSC. It has been shown that has-miR-144-3p was related to metastasis and malignant transformation in LUSC, whereas staging, poor survival, and differentiation of LUSC were always linked with has-miR-30e-3p (Figure 8F, 8G). However, there is no evidence suggesting that has-mir-144-3p and has-mir-30e-3p regulate POSTN expression in LUAD. As shown in Supplementary Figure 3, has-mir-30e-3p levels were similar between LUAD and control healthy samples. For has-mir-144-3p, there is no significant difference in the correlation and the prognosis between the POSTN and POSTN-targeting miRNAs in LUAD and control healthy samples. Therefore, has-miR-144-3p and has-miR-30e-3p may be the most clinically significant as these were the only miRNAs which are related to poor prognosis in LUSC.

## DISCUSSION

The mortality rate of lung cancer was about 75% in the United States at the beginning of the 20th century [28]. Although it is estimated that the 5-year survival rate of NSCLC was increased from 40.2% in 2014–2018 to 52.7% in 2019–2023 in China [29, 30], it is often diagnosed in advanced stages with a poor prognosis. Thus, there is an urgent need to elucidate the mechanism of lung cancer to identify potential therapeutic targets or prognostic biomarkers. In this study, the pan-cancer analysis revealed that POSTN was upregulated in lung cancer patients with poor prognoses. The level of POSTN was associated with clinicopathological parameters, including lymph node metastases and TNM stage. Furthermore, there was a close correlation between the level of POSTN and immune infiltration in lung cancer, especially in LUSC. In light of survival curves, we confirm that POSTN can be used as a promising therapeutic biomarker. Moreover, the upstream regulatory mechanism of POSTN in LUSC was investigated here to further study the mechanism of lung cancer.

POSTN is an ECM moiety and an adhesion molecule and has been found to promote tumour growth and metastasis [31, 32]. Overexpression of POSTN is correlated with poor overall survival in multiple cancers. High-level expression of POSTN can promote EMT via ILK/AKT/mTOR pathway in renal carcinoma [33]. Additionally, POSTN plays a significant role in chemoresistance during cancer treatment. A synthetic anti-POSTN peptide with binding affinity to POSTN has been shown to downregulate POSTN, thereafter reverse the drug resistance to doxorubicin in POSTN-overexpressed breast cancer cells [34]. Furthermore, the knockdown of POSTN was negatively correlated with the abilities of hepatocellular carcinoma cells to form tumours in mice [35]. In the current study, we analysed the expression of POSTN in various lung cancers using the Oncomine database and Timer database. The results are consistent with those reports that POSTN was up in several tumour tissues, including lung cancer tissues. However, the prognostic significance of POSTN in lung cancer patients is largely unclear. Our results showed that the overexpression of POSTN was associated with poor OS and PFS in lung cancer. In addition, high level of POSTN was positively related to lymph node metastases and the TNM stages. Based on our findings, POSTN can be a promising prognostic biomarker for lung cancer.

In lung cancer the start of the disease is thought to be a failure of the recognition of the cancerous cells by the immune system. The progression of lung cancer is also strongly related to the infiltration of immune cells, and our study now shows that Periostin is critical in this process, especially in neutrophil-dominant and macrophage-dominant inflammation [36]. Therefore, the upregulation of Periostin may facilitate immune cell inflammation in lung cancer. Furthermore, the TME is the internal environment not just for tumour cells to generate and develop, but also other tumour supporting cells, such as immune cells and fibroblasts fibroblasts, which can induce the release of IL-6 by activating YAP-TAZ pathway, as well as NF-κB and TNF-α, thereby contributing to immune cell infiltration and promotion of tumor progression [37]. Therefore, it is important for tumour development, invasion, and metastasis. The annual report on tumor progress released by the American Society of Clinical Oncology in 2021 points out that it is necessary to identify blood and tissue biomarkers related to immunotherapy, which is helpful for the design of tumor immunotherapy [38–40]. The biomarker related to immunotherapy is helpful for the design of tumour immunotherapy, which targets the immune microenvironment. Thus, the heterogeneity of the tumour immune microenvironment determines the efficacy of given immunotherapy. Therefore, the immune microenvironment is expected to guide clinical treatment and screen for patients who can benefit from immunotherapy. However, it is a great challenge to find good biomarkers to effectively predict the efficacy of immunotherapy [41].

Here, we found that POSTN can regulate TME. Several common hub genes related to POSTN in LUAD and LUSC were identified to be involved in the process of tumour development and immune response, which were confirmed by GO analysis and pathway enrichment analysis, including ECM-receptor interaction, the intestinal immune network for IgA production, cell-substrate adhesion, and integrin-mediated signalling pathway. Moreover, immune cells infiltration in TME, as well as the expression of immune checkpoints, has a significant impact on the development and metastasis of lung cancer [42, 43]. Here, we confirmed that the overexpression of POSTN was correlated with several immune cell infiltration in lung cancer tissues, including B cells, CD8+ T cells, CD4+T cells, macrophages, neutrophils, and dendritic cells. Moreover, increased CD274, PDCD1, and CTLA4 levels are strongly related to the overexpression of POSTN in lung cancer, especially LUSC. We further analysed the immune cell markers in LUAD and LUSC. There was a significant correlation between POSTN and several immune cell markers after cell purity correction. These findings indicated that immune cell infiltration exerts an important role in the development, invasion, and metastasis of lung cancer, especially LUSC, which can also be confirmed by the connections between POSTN level and several immune signatures, including lymphocytes, immunomodulators, and chemokines. Overexpression of POSTN may affect the prognosis of LUSC patients through immune cell infiltration. Thus, POSTN can be identified as the potential target of immunotherapy in lung cancer.

Overexpression of POSTN is related to immune infiltration significantly in LUSC patients. miRNA is documented to participate in the regulation of gene expression [43]. Thus, to further clarify the downregulated mechanisms of POSTN in LUSC, we investigated several common POSTN-target miRNAs using multiple databases. The results showed that those miRNAs may potentially bind to POSTN; however, only has-miR-144-3p and has-miR-30e-3p were significantly related to poor prognosis in LUSC patients. It has been reported that has-miR-144-3p was related to metastasis and malignant transformation in LUSC [44–46]. We confirmed that the overexpression of POSTN can be identified as the target to predict shorter survival times in LUSC. In addition, has-miR-30e-3p was downregulated in LUSC tissues, which was proved to be strongly related to staging, poor survival, and differentiation [47–50]. However, there is no evidence suggesting that has-mir-144-3p and has-mir-30e-3p regulate POSTN expression in LUAD. All the results help us understand the working mechanisms of POSTN in LUSC. ROC curve analysis further confirmed the clinical prognostic value of POSTN, where POSTN had a significantly high AUC value in the detection of LUSC, with high sensitivity and specificity. These results supported the proposal that POSTN can be a promising biomarker for poor prognosis in LUSC patients.

In summary, we systematically investigated the diagnostic and prognostic significance of POSTN in lung cancer. The overexpression of POSTN was strongly correlated with the immune cell infiltration in lung cancer patients with poor prognoses. In addition, we identified the upstream POSTN-regulating miRNAs in LUSC. All findings suggest POSTN can be a promising novel and sensitive biomarker for prognosis and immunotherapeutic target in patients with LUSC. However, all these results need to be validated by *in vitro* and *in vivo* experiments before clinical trials in the future.

## Supplementary Materials

Supplementary Figures

Supplementary Table 1
