# Supplementary material for: miRNAs-mediated overexpression of Periostin is correlated with poor prognosis and immune infiltration in lung squamous cell carcinoma
Source: Aging (Albany NY). 2022 May 4;14(9):3757–81. doi: 10.18632/aging.204056 (PMC9134939; doi:10.18632/aging.204056)
Supplement: Supplementary Figures [file aging-14-204056-s001.pdf]

SUPPLEMENTARY FIGURES

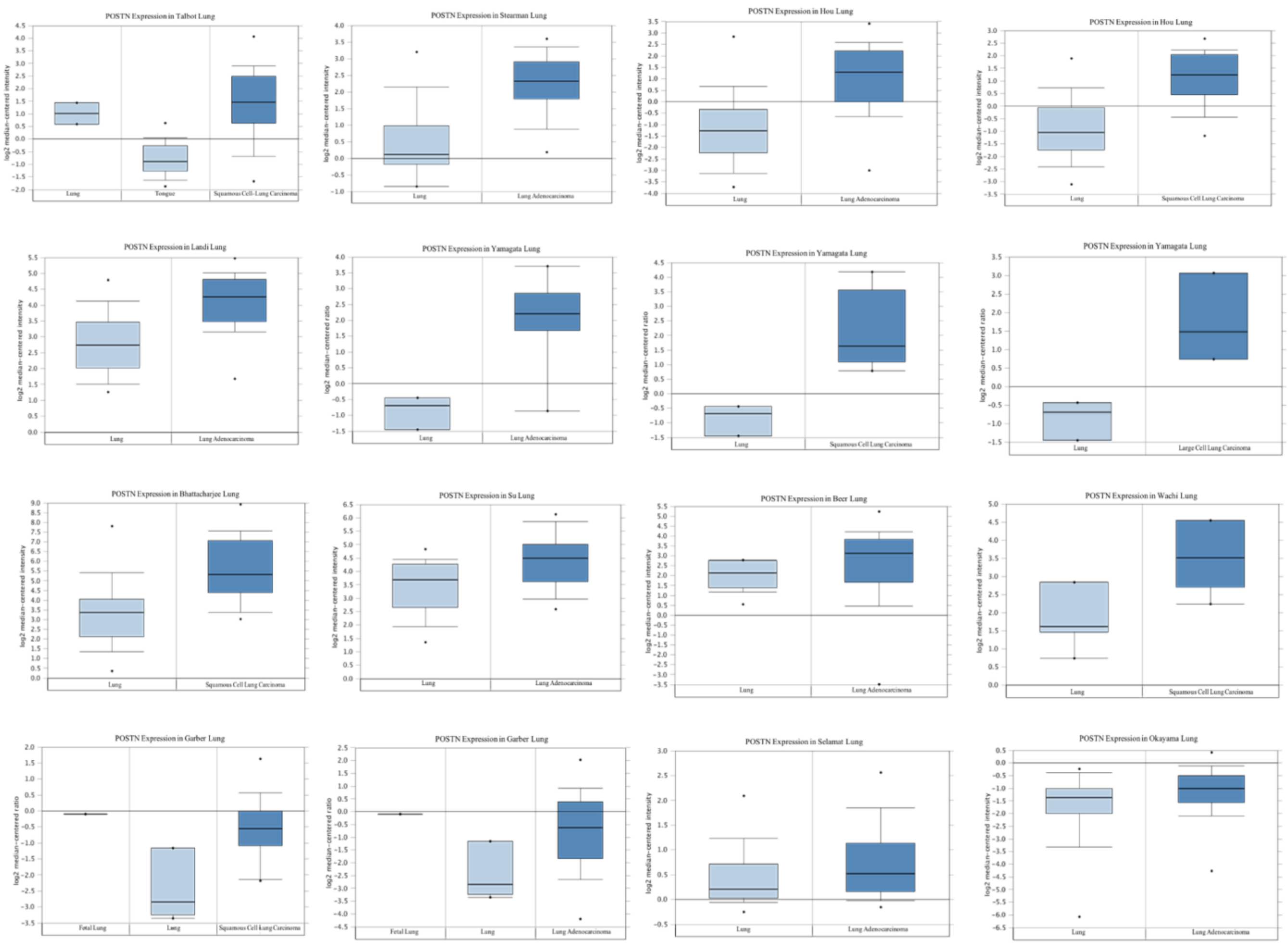

Supplementary Figure 1. POSTN expression in different types of lung cancer patients and normal individuals from the Oncomine database.

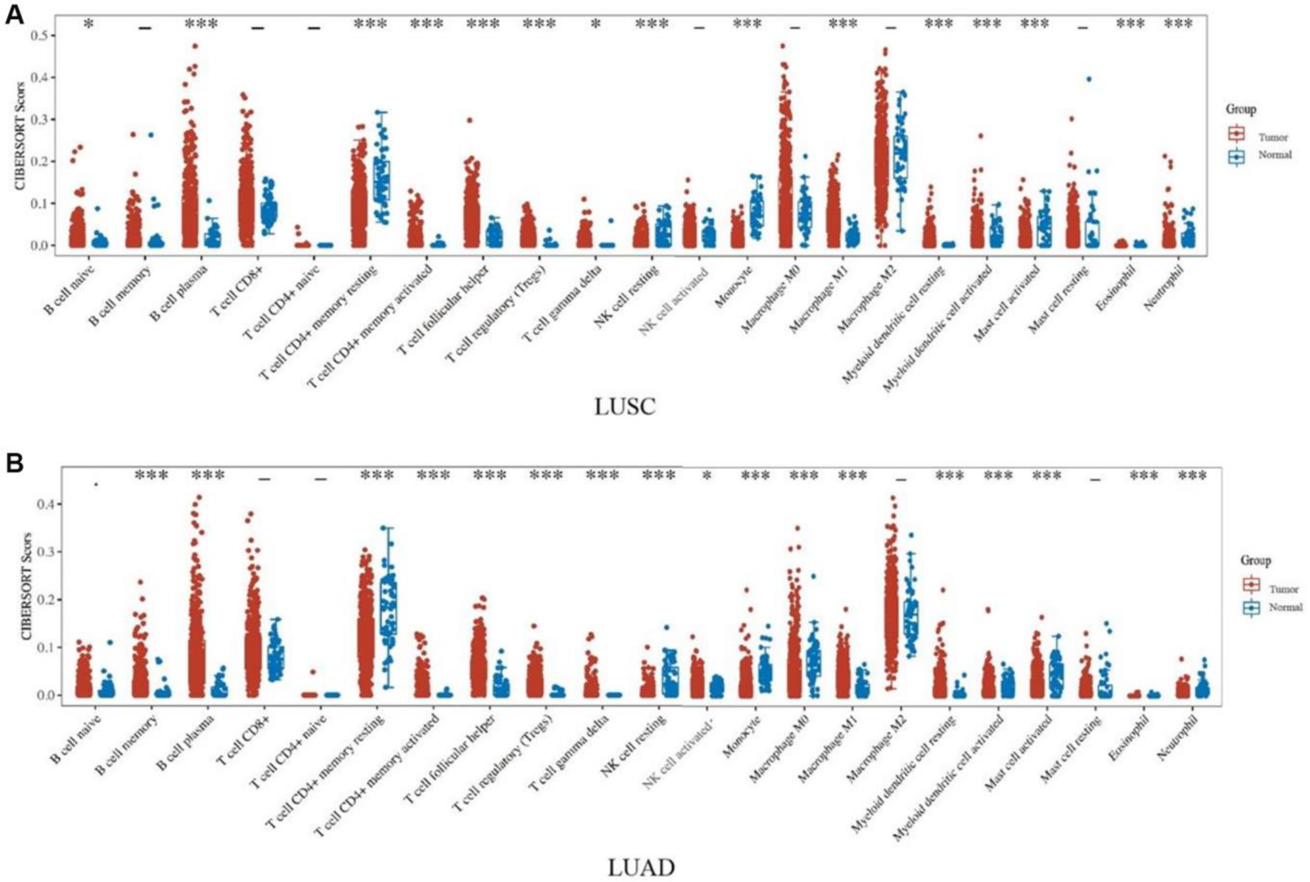

Supplementary Figure 2. Immune cell infiltration differs between tumor and normal tissues. The differences of POSTN expression in immune cell subsets in LUSC and normal groups (**A**). The differences of POSTN expression in immune cell subsets in LUSC and normal groups (**B**). $^{*}p < 0.05$, $^{**}p < 0.01$, $^{***}p < 0.001$.

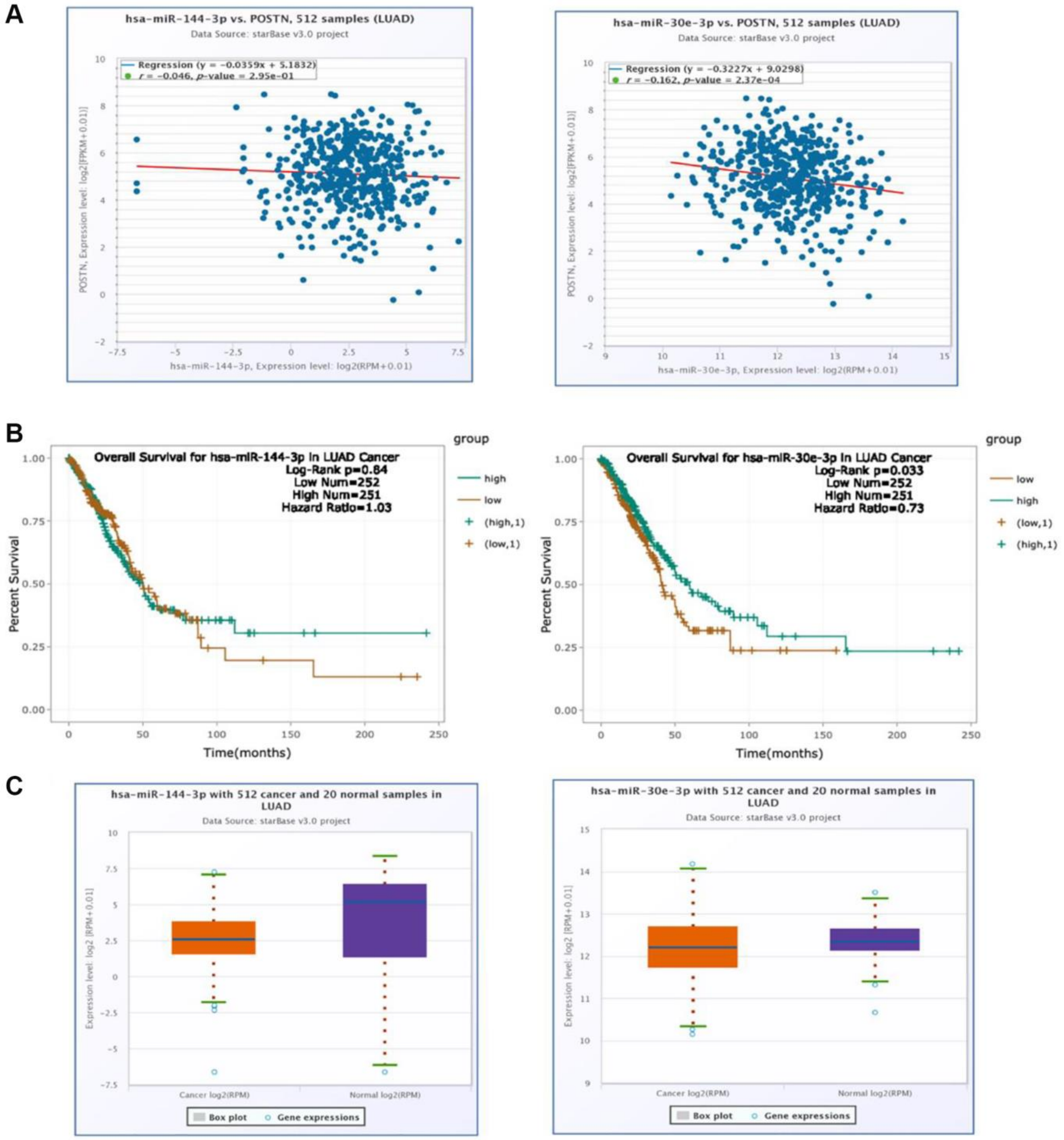

Supplementary Figure 3. Diagnostic value of POSTN in LUAD. The correlation between the expression of POSTN and POSTN-targeted miRNAs in LUAD and control normal samples determined by the starBase database (**A**). The prognostic value of POSTN-targeted miRNAs in LUAD assessed by Kaplan-Meier plotter (**B**). The expression of POSTN-targeted miRNAs in LUAD and control normal samples determined by starBase database (**C**).
