# Supplementary material for: miRNAs-mediated overexpression of Periostin is correlated with poor prognosis and immune infiltration in lung squamous cell carcinoma
Source: Aging (Albany NY). 2022 May 4;14(9):3757–81. doi: 10.18632/aging.204056 (PMC9134939; doi:10.18632/aging.204056)
Supplement: Supplementary Table 1 [file aging-14-204056-s002.pdf]

SUPPLEMENTARY TABLE

Supplementary Table 1. POSTN expression in different subtypes of lung cancer and normal tissues using the Oncomine database.

	Type of lung cancer versus normal lung tissue	*P*-value	*t*-Test	Fold change
Talbot Lung	Squamous Cell Lung Carcinoma	2.23E-10	7.588	4.315
Stearman Lung	Lung Adenocarcinoma	1.36E-06	5.557	3.313
Hou Lung	Lung Adenocarcinoma	1.12E-12	8.025	4.546
	Squamous Cell Lung Carcinoma	1.52E-11	8.285	4.087
Landi Lung	Lung Adenocarcinoma	2.83E-12	7.895	2.499
Yamagata Lung	Lung Adenocarcinoma	3.12E-04	5.102	6.546
	Squamous Cell Lung Carcinoma	1.54E-04	5.886	7.14
	Large Cell Lung Carcinoma	0.023	3.484	6.157
Bhattacharjee Lung	Squamous Cell Lung Carcinoma	1.10E-04	4.133	5.158
Su Lung	Lung Adenocarcinoma	1.21E-04	3.931	2.017
Beer Lung	Lung Adenocarcinoma	0.018	2.244	1.587
Wachi Lung	Squamous Cell Lung Carcinoma	0.008	3.061	3.18
Garber Lung	Squamous Cell Lung Carcinoma	0.014	2.654	3.011
	Lung Adenocarcinoma	0.023	2.442	2.63
Selamat Lung	Lung Adenocarcinoma	0.01	2.373	1.215
Okayama Lung	Lung Adenocarcinoma	0.027	2.052	1.547
